# Corneal endothelial integrity in aging mice lacking superoxide dismutase-1 and/or superoxide dismutase-3

**Published:** 2008-11-07

**Authors:** Anders Behndig

**Affiliations:** Department of Clinical Science/Ophthalmology, Umeå University Hospital, Umeå, Sweden

## Abstract

**Purpose:**

To evaluate the age-induced changes in corneal endothelial morphology in mice lacking the cytosolic copper-zinc superoxide dismutase (SOD-1), the interstitial extracellular superoxide dismutase (SOD-3), or both of these SOD isoenzymes.

**Methods:**

The central corneal endothelial morphologies of old C57BL-6J wild type (n=19), *SOD-1* null (n=16), *SOD-3* null (n=15), and *SOD1/3* null (n=11) mice were evaluated using alizarin red staining and light microscope photographs. For comparison, young endothelia from the same genotypes were evaluated similarly. The levels of corneal reactive oxygen species and nitrogen species in all four genotypes were quantified using fluorimetry with 2',7'-dichlorodihydrofluorescein diacetate and OxyBURST.

**Results:**

In accordance with our previous findings, the mean corneal endothelial cell area was larger in the *SOD-3* null genotype than in the wild type mice. The *SOD-1/3* null genotype had similar cell sizes as the *SOD-3* null mice but had a more irregular morphology at an older age. Apparently, these irregularities develop with time as they are not seen in young animals. The *SOD-1* null mice did not differ from the wild type mice in corneal endothelial morphology. Elevated levels of reactive oxygen species were seen in *SOD-1* null and *SOD-3* null corneas, and elevated superoxide levels were seen in all three knockout genotypes.

**Conclusions:**

The increased spontaneous age-related enlargement of corneal endothelial cells seen in the absence of SOD-3 is associated with a more irregular cell pattern when combined with a lack of SOD-1. This indicates more cellular movements and ongoing repair in the *SOD-1/3* null genotype and possibly a more vulnerable corneal endothelium. SOD-3 and SOD-1 appear to have functions in preserving corneal endothelial integrity in aging.

## Introduction

The monolayer of corneal endothelial cells is of great importance to maintain corneal dehydration, tissue integrity, and transparency. The corneal endothelial cell density decreases due to a continuing cell loss throughout life [1,2], which is compensated for by the sliding of adjacent cells to cover any defect in the endothelial cell layer. Thus, in a human being, the average corneal endothelial cell size increases from 200–250 µm^2^ in childhood to 400–700 µm^2^ in adults. We have previously shown that a similar cell enlargement takes place also in the mouse from 240±49 µm^2^ at the age of one month to 429±99 µm^2^ at one year and 517±209 µm^2^ at two years of age in the C57BL-6J strain [3]. Loss of corneal endothelial cells below a critical level will lead to corneal decompensation with edema [4], a well recognized clinical problem seen in Fuchs’ endothelial dystrophy [5] and bullous keratopathy, both major causes for corneal transplantation. Corneal endothelial cell loss generally occurs via apoptosis [4,5] and has repeatedly been associated with increased oxidative stress [6-9].

Although corneal endothelial cells have some degree of mitotic capacity, at least in lower mammals [10-13], they generally do not divide in resting conditions [14]. In stress conditions, however, such as surgery [12,15,16], trauma [17,18], and inflammation [19-21], endothelial cells will divide in lower mammals [14]. In higher and lower mammals, the cells will show a deviation from their normal, uniform, hexagonal cellular pattern [2,12,22] in stress conditions due to an increased cellular sliding and movement in the healing endothelium.

Interstitial extracellular superoxide dismutase (SOD-3) [23] has a high affinity for sulfated glycosaminoglycans (GAGs) and is attached to proteoglycans in the intercellular matrix as well as on cell surfaces [24,25]. SOD-3 is a major superoxide dismutase isoenzyme in the human cornea [26]. The presence of SOD-3 has been demonstrated by immunohistochemistry in the murine corneal endothelium [3] and has a role in preserving the corneal endothelial integrity in aging and acute inflammatory injuries in mice [3]. Copper-zinc superoxide dismutase (SOD-1), located in the cytosol and the mitochondrial intermembranous space, has also been demonstrated similarly in the corneal endothelium in mice [3] and in man [27], but its possible role for endothelial viability in aging has not been investigated.

In the present investigation, we study the effects of the absence of these two SOD isoenzymes on corneal endothelial morphology in normal aging in a murine knockout model using three knockout strains and C57BL-6J wild type mice as controls. In addition, we quantified the levels of corneal reactive oxygen species and nitrogen species in all four genotypes using fluorimetric methodology. The strains investigated are *SOD-1* null, *SOD-3* null and *SOD-1/3* null, the latter lacking both *SOD-1* and *SOD-3*.

## Methods

### Animals

The investigation adhered to the ARVO Statement for the Use of Animals in Ophthalmic and Vision Research and was approved by the research ethics committee of Umeå University (Umeå, Sweden). The CuZn-*SOD* null mice (initial background 129/CD1) were generated by Dr. A. G. Reaume et al. [28] at Cephalon Inc. (Frazer, PA). Female CuZn-*SOD* null mice are essentially sterile so breeding was accomplished with heterozygotic female mice. The genotype of each offspring was determined. Two methods were used for determining the genotype. First, polymerase chain reaction (PCR) primers were designed to specifically recognize the genes, one pair for genomic *SOD-1* and one for the inserted neomycin gene. To distinguish between the homozygous and heterozygous *SOD-1* null genotype, the SOD-1 protein content in erythrocytes were determined using an ELISA. Sixteen *SOD-1* null mice of mixed genders aged 64.9±22.3 weeks (mean±SD) and three *SOD-1* null mice aged 12.1 weeks were used for endothelial morphology. In addition, 11 *SOD-1* null mice aged 13.8±0.9 weeks were used for reactive oxygen species/reactive nitrogen species (ROS/RNS) quantification. The *SOD-3* null mice (background C57BL/6×129/SV) were obtained from a breeding colony established at Umeå University [29]. Fifteen *SOD-3* null mice of mixed genders aged 57.5±27.0 weeks and three *SOD-3* null mice aged 10.9±3.0 weeks were used in endothelial morphology, and 11 *SOD-3* null mice aged 13.7±2.5 weeks were used for ROS/RNS quantification. The *SOD-1/3* null strain was obtained by cross-breeding mice heterozygotic for *SOD-1* with *SOD-3* null mice. The genotype of each offspring was determined as described above for the *SOD-1* null mice. Eleven *SOD-1/3* null mice of mixed genders aged 49.9±5.8 weeks and three *SOD-1/3* null mice aged 11.8±2.1 weeks were used for endothelial morphology, and eight *SOD-1/3* null mice aged 16.1 weeks were used for ROS/RNS quantification. All three knockout strains were backcrossed 10 times into C57BL-6J. Nineteen wild type C57BL-6J mice aged 61.8±18.5 weeks and three aged 10.4±3.0 weeks were used as controls for endothelial morphology, and 12 mice of the same genotype aged 15.0±1.6 weeks were used as controls for ROS/RNS quantification.

### Corneal endothelial morphology

All animals were killed with cervical dislocation, and both corneas were dissected. After making peripheral radial cuts, the specimens were placed on glass slides, and the endothelium was stained with alizarin red S (Sigma- Aldrich, Inc. St. Louis, MO) [30]. The central part of the endothelium was photographed in a light microscope at 400X magnification, and the digital photos were analyzed using the ImageJ image analysis program (National Institutes of Health, Bethesda, MD). Four specimens (two wild type, one *SOD-3* null and one *SOD1/3* null) were excluded due to dissection or preparation artifacts. Cell area (A), perimeter (P), maximal inertia moment (I_max_), and minimal inertia moment (I_min_) were determined for a central cluster of 55 cells in each specimen by marking the cell corners manually [3,31,32]. The degree of elongation (DE) of cells was calculated as (I_max_-I_min_)/(I_max_+I_min_) [12], and the deviation from the ideal hexagonal cell shape (hexagon shape factor, HSF) was calculated as abs (P^2^/A-13.856) as previously described [3]. The cell polymegethism was quantified using the coefficient of variation of cell size (CV), which was calculated as the standard deviation of the cell sizes for a specimen divided by the mean cell size for the same specimen.

### Quantification of corneal reactive oxygen species

The levels of reactive oxygen species (ROS) and reactive nitrogen species (RNS) in corneal specimens were quantified using two fluorimetric probes, 2',7'-dichlorodihydrofluorescein diacetate (H_2_DCFDA; Invitrogen, Inc., Carlsbad, CA), which detects hydrogen peroxide, peroxyl radicals, and peroxynitrite, and the amine-reactive green-dye assay, OxyBURST Green (Invitrogen), which detects superoxide anion radicals. After dissection, the corneal specimens were washed with PBS and immediately incubated on 96 well plates in 200 µl PBS for 30 min at 37 °C. The background fluorescence for each specimen was determined with a fluorimeter (excitation=488 nm, emission=520 nm). After this, H_2_DCFDA or OxyBURST Green was added to each well to a final concentration of 10 µM. The plates were again incubated for 30 min at 37 °C, and the fluorescence was measured similarly. The ROS/RNS and superoxide levels were calculated as the fluorescence after subtraction of the background fluorescence.

Student’s two-tailed *t*-test was used for statistical analysis, using the appropriate Bonferroni corrections for comparisons of multiple groups, and p<0.05 was considered statistically significant.

## Results

**Figure 1:**
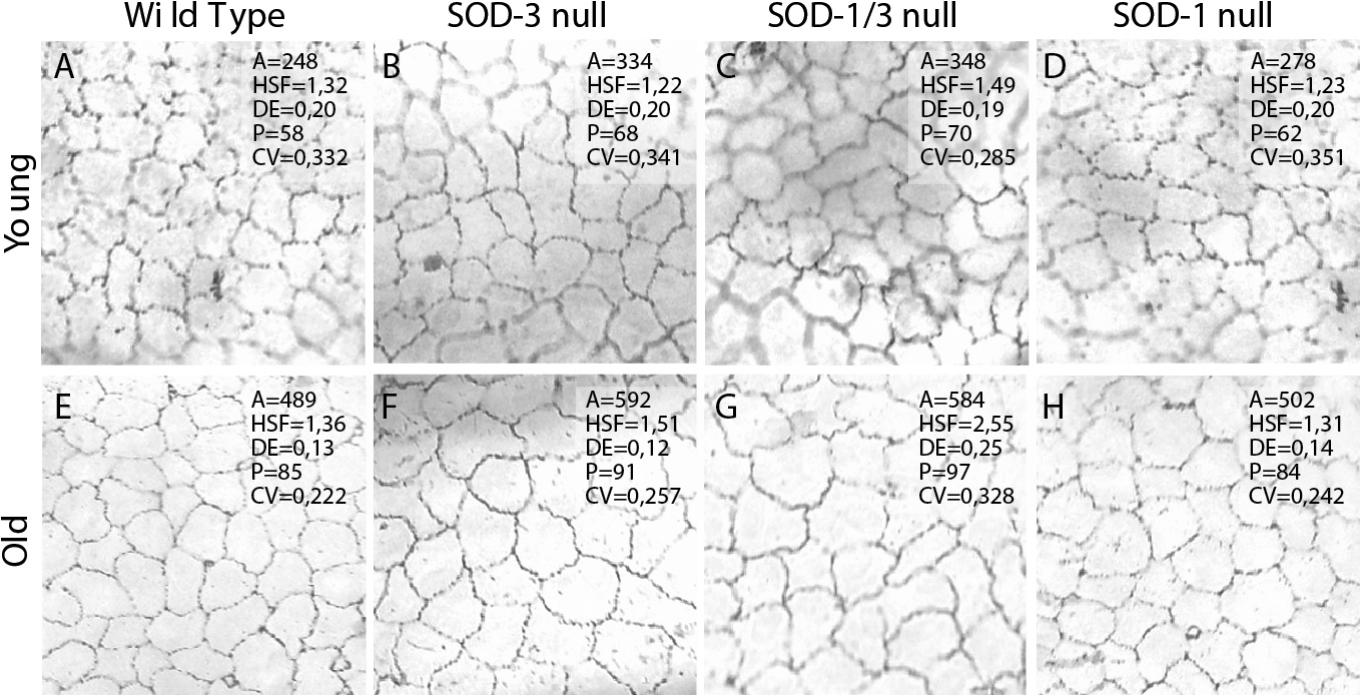
Corneal endothelial photographs. **A**: Typical corneal endothelial photograph with alizarin red staining from a C57BL/6 wild type mouse at a young age. A=mean cell area, HSF=hexagon shape factor, quantifying the deviation from the ideal hexagonal cell shape; DE=degree of cell elongation; P=mean cell perimeter. **B**: SOD-3 null mouse, young age. The cells are enlarged, compared to the wildtype control. **C**: SOD-1/3 null mouse, young age. The cells are enlarged, compared to the wildtype control. **D**: SOD-1 null mouse, young age. **E**: Wild type mouse, old age. **F**: SOD-3 null mouse, old age. The cells are enlarged, compared to the wildtype control. **G**: SOD-1/3 null mouse, old age. Note that the cells are enlarged and show increased pleomorphism and polymegethism, compared to the wildtype control. **H**: SOD-1 null mouse, old age.

The mean corneal endothelial cell area (A) and perimeter (P) were larger in *SOD-3* null mice than in wild type mice in both young animals (p=0.030 and p=0.027, respectively) and old animals (p<0.001; Figure 1; Table 1). The other morphological variables did not differ between the *SOD-3* null specimens and the wild type controls. In the *SOD-1/3* null mice, the mean cell area was similar to that in the *SOD-3* null mice (p=n.s.; Table 1), but in the old mice, the *SOD1/3* null endothelia showed a larger cell perimeter (p=0.0074), were more elongated (p<0.001) and irregular (p<0.001), and showed a more pronounced polymegethism (p<0.001; Figure 1; Table 1) than in the *SOD-3* null specimens. In the few younger mice, no difference in cell shape or cell size could be demonstrated between the *SOD-3* null and *SOD-1/3* null mice. The *SOD-1* null mice did not differ from the wild type controls in corneal endothelial morphology in the young or old.

**Table 1:** Summary of corneal endothelial morphology at older ages in mice lacking superoxide dismutase 1 and/or 3 and in C57BL/6 wild type controls.

**Mouse type**	**Age (months)**	**A (µm^2^)**	**HSF (A.U.)**	**DE (A.U.)**	**P (µm)**	**CV**	**n=**
**Wild type**	10.4±3.0	246±35	1.30±0.40	0.19±0.02	58±4	0.35±0.027	6
***SOD-3* null**	10.9±3.0	353±56*	1.35±0.23	0.19±0.02	69±6*	0.35±0.064	6
***SOD-1/3* null**	11.8±2.1	360±95*	1.39±0.20	0.19±0.02	69±9*	0.30±0.032	6
***SOD-1* null**	12.1±0.0	273±25	1.35±0.24	0.19±0.02	61±3	0.36±0.018	6
**Wild type**	61.8±18.5	494±57	1.30±0.32	0.14±0.02	84±5	0.24±0.036	36
***SOD-3* null**	57.5±27.0	637±121*	1.45±0.29	0.14±0.02	91±8*	0.23±0.051	29
***SOD-1/3* null**	49.9±5.8	619±67*	2.21±0.75*	0.19±0.06*	98±6*#	0.31±0.057*#	21
***SOD-1* null**	64.9±22.3	504±74	1.36±0.27	0.14±0.02	83±6	0.26±0.038	32

A=mean cell area; HSF=hexagon shape factor (quantifying the deviation from the ideal hexagonal cell shape); DE=degree of cell elongation; P=mean cell perimeter; CV= coefficient of variation, expressing the degree of cell polymegethism; A.U.=arbitrary units. The asterisk indicates that there was significant difference from the corresponding wild type control, and the sharp (hash mark) signified that there was significant difference from the *SOD-3* null genotype. The absence of SOD-3 leads to larger cells, and in the absence of SOD-3 and SOD-1, the cells also become more irregular with increased pleomorphism and polymegethism as the mice age.

**Figure 2:**
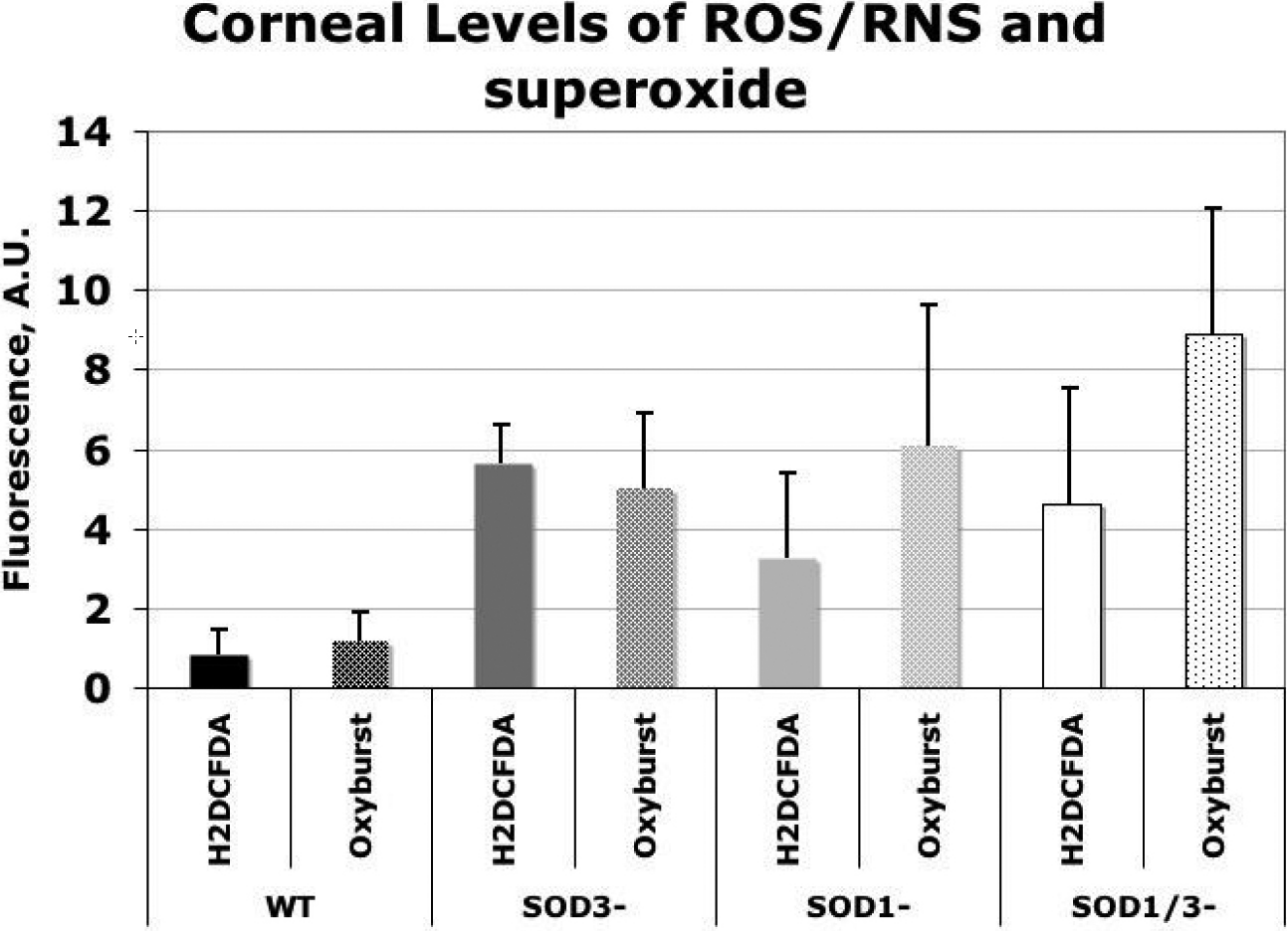
Levels of corneal ROS/RNS and superoxide radicals in C57BL/6 wild type, *SOD-3* null, *SOD-1* null, and *SOD-1/3* null mice expressed as arbitrary units. The ROS/RNS levels were quantified using fluorimetry with 10 µM 2',7'-dichlorodihydrofluorescein diacetate (H_2_DCFDA), and the superoxide levels were determined similarly with 10 µM OxyBURST Green dye assay (excitation=488 nm, emission=520 nm). Both the ROS/RNS and superoxide levels are significantly elevated in all three knockout genotypes (p<0.05), except for the ROS/RNS levels in the *SOD-1/3* null genotype (p=0.078). WT, wild type; A.U., arbitrary units.

The levels of ROS/RNS and superoxide were significantly elevated in all three knockout genotypes compared to the wild type controls, except for the ROS/RNS levels in the *SOD-1/3* null genotype where the difference did not reach statistical significance, which is likely due to the small number of specimens (Figure 2).

## Discussion

This paper confirms that the absence of SOD-3 results in a decreased corneal endothelial cell viability in normal aging [3], suggesting that SOD-3 is of importance for preserving corneal endothelial integrity. Furthermore, when combining an absence of SOD-3 with an absence of SOD-1, a more irregular endothelial cell pattern develops with time with increased pleomorphism and polymegethism. Similar endothelial irregularities can be seen when the endothelium is in repair phase after an injury [2,12,22], a common example in humans is after routine cataract surgery [32]. In such acute injuries, the endothelial changes are often accompanied by an acute but reversible corneal edema [32]. The *SOD-3* null and *SOD1/3* null mice in the present study did not develop a spontaneous corneal edema at an older age despite the morphological corneal endothelial changes. This likely owes to the large functional reserve of the corneal endothelium [1,33]. The endothelial response to an acute oxidative injury in the absence of SOD-3 and SOD-1 will be the subject of an upcoming investigation.

In the present study, we also demonstrate elevated levels of ROS/RNS in the cornea of the *SOD-1* null and *SOD-3* null genotypes and elevated superoxide radical levels in all three knockout genotypes using fluorimetry. Similarly, we have previously demonstrated elevated levels of extracellular superoxide radicals in the murine cornea in *SOD-3* null mice using luminometry with 25 µM lucigenin [3] and increased superoxide levels in the absence of SOD-1 in the murine lens [34,35]. These results show that the ocular tissues are exposed to augmented oxidative stress in the absence of superoxide dismutase.

Taken together, the findings of this study suggest that the superoxide radical and oxidative stress contribute to age-dependent corneal endothelial cell loss. An accelerated age-dependent endothelial cell loss through apoptosis is seen in humans such as in Fuchs’ endothelial dystrophy [5] where indications for an involvement of oxidative stress have been demonstrated [9]. Similarly, photooxidative injury induces corneal endothelial cell apoptosis in animal models [36,37]. The present study uses knockout mouse models to indicate that reduced scavenging of O_2_^-^**·** may be involved in age-related corneal endothelial cell death.

Altered endothelial cell morphology with cell elongation and pleomorphism are more commonly seen in acute corneal injuries [12,13,15,17,18], but in the present investigation, we did see some changes in endothelial cell shape in the absence of both SOD-1 and SOD-3, and these changes were not seen in the absence of just one of these isoenzymes. SOD-1 and SOD-3 scavenge intracellular and extracellular O_2_^-^**·** radicals, respectively, and the functions of these isoenzymes are therefore not interchangeable [38,39]. The present findings indicate more cellular movement and ongoing repair in the *SOD-1/3* null genotype and possibly a more vulnerable corneal endothelium in the absence of both these SOD isoenzymes. A plausible explanation for these findings could be additive effects from extracellular and intracellular oxidative stress [39]. To conclude, both SOD-3 and SOD-1 appear to have functions in preserving corneal endothelial integrity in aging.
